# Venous thromboembolism risk in psychiatric in-patients: a multicentre cross-sectional study

**DOI:** 10.1192/bjb.2019.25

**Published:** 2019-12

**Authors:** Natalie Ellis, Carla-Marie Grubb, Sophie Mustoe, Eleanor Watkins, David Codling, Sarah Fitch, Lucy Stirland, Munzir Quraishy, Josie Jenkinson, Judith Harrison

**Affiliations:** 1 Cardiff University Medical School; 2 King's College London Medical School; 3 Edinburgh University Medical School; 4 King's College NHS Foundation Trust; 5 Cardiff and Vale University Health Board; 6 University of Edinburgh; 7 South West London and St George's Mental Health NHS Trust; 8 Cardiff University

**Keywords:** Quality improvement, venous thromboembolism, psychiatric in-patients

## Abstract

**Aims and method:**

We assessed venous thromboembolism (VTE) risk, barriers to prescribing VTE prophylaxis and completion of VTE risk assessment in psychiatric in-patients. This was a cross-sectional study conducted across three centres. We used the UK Department of Health VTE risk assessment tool which had been adapted for psychiatric patients.

**Results:**

Of the 470 patients assessed, 144 (30.6%) were at increased risk of VTE. Patients on old age wards were more likely to be at increased risk than those on general adult wards (odds ratio = 2.26, 95% CI 1.51–3.37). Of those at higher risk of VTE, auditors recorded concerns about prescribing prophylaxis in 70 patients (14.9%). Only 20 (4.3%) patients had a completed risk assessment.

**Clinical implications:**

Mental health in-patients are likely to be at increased risk of VTE. VTE risk assessment is not currently embedded in psychiatric in-patient care. There is a need for guidance specific to this population.

Venous thromboembolism (VTE) is a potentially fatal condition. Hospital-associated VTE leads to more than 25 000 deaths per year in the UK.^1^ VTE-related morbidity has significant effects on quality of life and healthcare costs.^2^ Extensive research has allowed the development of guidelines for diagnosis and management of VTE risk in hospitals.^3^ However, there is a paucity of evidence regarding VTE risk in psychiatric in-patients. In March 2018, the National Institute for Health and Care and Excellence (NICE) released updated guidelines for VTE.^4^ They included a new recommendation that all mental health in-patients should have a VTE risk assessment on admission.

VTE has an incidence of between 2 and 12% among psychiatric in-patients.^5,6^ While psychiatric in-patients are often more mobile than those in acute hospital wards, there is evidence of psychiatry-specific risk factors. Case reports have linked VTE with both antipsychotic drugs^7–10^ and physical restraint.^11–14^ Antipsychotics can increase risk of VTE 3–4-fold.^7^ Prospective cohort studies have found that patients who had been physically restrained were more likely to develop VTE.^5^ Patients with a diagnosis of dementia are also likely to develop VTE.^6^ Additionally, many psychiatric in-patients are aged over 60 years or have comorbidities which increase their risk of VTE.^15–17^

This cross-sectional, multicentre study aimed to assess VTE risk in psychiatric in-patients. We hypothesised that: (a) a significant proportion of psychiatric patients are at risk of VTE, and (b) VTE risk is not routinely assessed in this group.

## Method

### Sample

Patient records were sampled from 27 psychiatric in-patient wards across three sites: Cardiff and Vale University Health Board, South London and the Maudsley NHS Foundation Trust, and NHS Lothian. The project was registered with the audit departments of each National Health Service (NHS) trust. As the project was under an audit framework, ethical approval was not required. Ten wards were included in Cardiff, nine in London and eight in Edinburgh. Addictions units and child and adolescent mental health services (CAMHS) wards were excluded.

### Data collection

Data were collected across the three sites by teams of medical students from Cardiff University, King's College London and the University of Edinburgh. Before commencing data collection, all students completed an online national training module on VTE assessment,^18^ ensuring they were knowledgeable about VTE risk and prophylactic management. The students were supervised locally by psychiatric trainees, as part of a larger nationwide student audit scheme, Student Psychiatry Audit and Research Collaborative.^19^

We used the UK Department of Health VTE risk assessment tool (available at https://www.nice.org.uk/guidance/ng89/resources/department-of-health-vte-risk-assessment-tool-pdf-4787149213), which had been adapted to include VTE risk factors thought to be specific for psychiatric patients (Table 1). These adaptations were based on the findings from a quality improvement programme conducted in South London and Maudsley NHS Foundation Trust in which semi-structured interviews were conducted with mental health staff. Information was gathered from electronic patient records and drug charts, with any discrepancies clarified with ward staff. A standardised electronic form was used to record data on the Welsh Digital Data Collection Platform.^20^ The assessors recorded whether each patient was at an increased risk of VTE based on the proforma and their clinical knowledge of VTE. They also noted whether the clinical team had recorded any perceived contraindications or other barriers to prescribing antithrombotic stockings or anticoagulants for the patient, and whether the patient had a completed VTE risk assessment form. The data were collected on 5–7 July 2016 in Cardiff, 12–19 December in London and 7–31 March 2017 in Edinburgh.

**Table 1:** Adapted Department of Health VTE risk assessment

Thrombosis risk	Bleeding risk	Patient characteristics
Presence of active cancer	Bleeding disorder	Psychiatric diagnosis
Age >60 years	Anticoagulated	Age
Dehydration	Previous stroke	Ethnicity
Thrombophilias	Thrombocytopenia	Antipsychotic use
Obesity	Hypertension	Physical restraint
Pregnancy	Recent neurosurgery	Thromboprophylaxis
Reduced mobility	Recent spinal procedures	Legal restrictions
Recent knee/hip replacement		
Recent hip fracture		
Recent surgical procedure		

### Statistical methods and data analysis

We used SPSS to conduct our data analysis. We calculated descriptive statistics for patient characteristics, risk assessment completion, patient VTE risk and documented reasons for not prescribing VTE prophylaxis. We conducted χ^2^ tests of independence to test for a difference between (a) VTE risk between the types of ward, (b) VTE risk between the different sites, (c) VTE risk assessment completion between the different sites, and (d) recorded concerns about prescribing VTE prophylaxis between the different sites.

### Ethics statement

This study came under the audit framework and so did not require formal ethical approval. The project was registered with the local audit department at each site and permission was granted.

## Results

### Patient characteristics

In total, the sample comprised 470 acute adult and old age psychiatric in-patients on 27 wards across the three sites: 195 patients in London, 130 in Edinburgh and 145 in Cardiff. Of these patients, 202 (43.0%) were over 60 years old. Table 2 outlines the percentage of patients with each primary diagnosis. A total of 365 (77.7%) patients were prescribed at least one antipsychotic medication, and eight (1.7%) patients had been physically restrained on that admission.

**Table 2:** Patients by diagnosis

Diagnosis	ICD-10 codes	Number of patients (%)
Functional psychosis	F20, F29, F23.2, F20.9	193 (41.1)
Bipolar affective disorder	F31	61 (13.0)
Depression/anxiety	F06.3, F06.4, F41, F41.0, F41.1, F41.2,	38 (8.1)
Neurodevelopmental disorder	F83, F84	4 (0.9)
Dementia	F00, F01, F02, F02.3, F03	98 (20.9)
Other organic disorder	F07.8, F07.9, F09	11 (2.3)
Drugs/alcohol	F10, F11, F12, F13, F14, F15, F16, F19, Z71.4	13 (2.8)
Other affective	F34.8, F38, F39	3 (0.6)
Personality disorder	F07.0, F60, F60.9	11 (2.3)
Other	F06.9, F99, Z71.1	38 (8.1)

### VTE risk

We found 144 (30.6%) of in-patients to be at an increased risk of VTE. Patients on old age wards were more likely to be at increased risk of VTE (41.4%) than patients on general adult wards (Table 3). In our sample, 96 (20.4%) patients had a diagnosis of dementia and 37 (39%) of these were found to be at an increased risk of VTE. The number of patients at an increased risk of VTE did not differ significantly between sites (*P* = 0.055).

**Table 3:** VTE risk

Patient characteristics	Number of patients (% of total)	Patients at risk of VTE according to tool (%)	*P* for independence (χ^2^)
All	470	144 (30.6)	
In-patients on old age wards	181 (38.5)	75 (41.4)	<0.001
In-patients on general adult wards	289 (61.5)	69 (23.9)	
Patients with dementia diagnosis	96 (20.4)	37 (39)	
Site: Cardiff	145 (30.9)	56 (38.6)	0.055
Site: Edinburgh	130 (27.7)	37 (28.5)	
Site: London	195 (41.5)	51 (26.2)	

### Risk assessment and contraindications

Of the 470 patients included, only 20 (4.3%) had a VTE risk assessment completed by the clinical team. There were significant differences among the sites in the proportion of patients who had a completed risk assessment form (Cardiff = 1.4%, London = 8.7%, Edinburgh = 0.8%, *P* < 0.001).

There were potential contraindications or concerns about prescribing VTE prophylaxis in 14.9% of all patients and 20.8% of those patients considered to be at increased VTE risk. Table 4 shows the perceived barriers to prophylaxis. There were significant differences among the three sites in the proportion of patients for whom there were concerns about prescribing VTE prophylaxis (Cardiff = 31%, London = 6%, Edinburgh = 10%, *P* < 0.001).

**Table 4:** Perceived barriers to prescribing VTE prophylaxis

Reason for concern	Number of patients (%)
Active self-harm	2 (0.43)
High risk of self-harm/previous self-harm	19 (4.04)
Self-neglect	25 (5.32)
Physical health risk	5 (1.06)
Medication	3 (0.64)
Alcohol use	1 (0.21)
Fall risk	6 (1.28)
‘Risk may outweigh benefits’	4 (0.85)
Multiple reasons stated	5 (1.06)

## Discussion

The primary aim of this study was to assess whether psychiatric in-patients are at increased risk of VTE. Nearly a third of patients were judged to be at increased risk, indicating that they would merit some form of intervention on an acute medical ward, such as increasing patient mobility, compression stockings or sub-cutaneous heparin. Our results support those of Choudry and Job,^21^ who audited VTE risk assessment on old-age psychiatric wards. They found that all patients assessed during the 2 week audit period had at least one risk factor for VTE, and nearly two-thirds of patients (63%) had at least three. None of the patients audited had received a risk assessment. Our findings are also supported by a large study in France, in which 458 psychiatric in-patients were followed for 90 days,^6^ and a study conducted in Japan (*N* = 190) where doppler ultrasound scanning was used to ensure that even asymptomatic VTE was recorded.^5^ Both of these studies found a high incidence of VTE among psychiatric in-patients. However, these studies did not record how many of these patients had received a risk assessment from ward staff before each study had started.

In our sample, the majority of patients were prescribed at least one antipsychotic medication. There is evidence to suggest a link between these drugs and VTE risk. Lacut and colleagues used a case–control study (*N* = 677) to examine the relationship between use of antipsychotic drugs and VTE.^7^ They demonstrated a 3.5-fold increased risk of VTE in patients exposed to antipsychotic agents, particularly phenothiazines, butyrophenones and benzamides. A strong association has been identified between chlorpromazine use and VTE, based on a cross-sectional study of more than 29 000 individuals who received antipsychotic drugs.^22^ The risk was greatest during the early stages of treatment. Clozapine has also been implicated in VTE risk, as it has been associated with a fatal pulmonary embolus rate of more than 27 times that seen in the general population.^8,9^ Comparison of such studies with those which have quantified risk for well-established VTE risk factors emphasise the importance of addressing psychiatric risk factors to reduce VTE incidence. For example, risk of VTE for women taking the combined contraceptive pill is almost three times that of non-exposed women,^23^ and the presence of malignant neoplasm has an odds ratio of 4.1 for VTE compared with those without cancer.^24^

In the current study, only a small minority of patients were exposed to frequent physical restraint. However, this factor should still be given consideration. Case reports have suggested that use of physical restraint is another psychiatric in-patient factor which may influence VTE risk.^12^ A significant association between physical restraint and the development of VTE has been demonstrated in patients with psychiatric illnesses.^25^ One study highlighted the effectiveness of prophylactic heparin in 170 patients with schizophrenia, 82% of whom underwent at least one episode of physical restraint.^26^ This was a large study (*N* = 679), although it had a retrospective design and all in-patients were from the same location, which reduces the generalisability of the results. VTE has also been shown to occur in physically restrained psychiatric patients despite pharmacological prophylaxis, suggesting that a thorough VTE assessment and multi-modal prophylaxis may be required in these patients.^5^ Mechanisms thought to be related to this association include stasis-induced vein wall injury and exaggerated endothelial tissue factor expression.^25^

We found that patients on old age psychiatric wards were at significantly greater risk of thromboembolism than those on general adult wards. The difference in the risk profile of these groups is not surprising given the overlap between thromboembolism risk and general ageing, including reduced mobility, as well as other specific comorbidities such as malignancy.^27^ In our study, almost 40% of those with dementia were at increased risk. Dementia may lead to VTE as it is associated with reduced mobility, frailty and dehydration.^28^ Moreover, impaired communication may delay diagnosis of VTE.

In addition to the medical contraindications to mechanical or pharmacological VTE prophylaxis, there may be other barriers to prescribing in mental health settings. Nearly a third (27%) of patients in our study were recorded as being at high risk of self-harm. Clinicians may be wary of prescribing compression stockings as they could be used as ligatures^29^ and anti-coagulant medications may increase bleeding in individuals who self-harm by cutting.

A minority of patients had a structured VTE risk assessment completed (4.3%) even when risk assessment proformas were readily available. In one centre, the VTE risk assessment proforma was incorporated into all drug charts. The low rates of proforma completion suggest that staff are not aware of the need to measure VTE risk. A higher proportion of patients at the London site had a VTE risk assessment than those in Cardiff or Edinburgh. A local audit of VTE risk had been completed at the London site previously, which may have resulted in greater awareness among staff. In March 2018, NICE released updated guidelines for VTE.^4^ They included a new recommendation that all mental health in-patients should have a VTE risk assessment on admission.

This study has several strengths. We studied a large sample from multiple centres and used a standardised assessment tool. The results should therefore be generalisable to other in-patient psychiatric populations. There are also some limitations. Some in-patient groups were not included, such as CAMHS and addictions units. The VTE risk assessment tool, while recommended by NICE, has not been formally validated in this population. However, we adapted it to include factors relevant to mental health. Assessors were required to judge whether each patient was at an increased risk of VTE based on the information collected. However, they had all undertaken additional training on VTE risk assessment and management, and were supervised by a doctor in psychiatry training. It would have been useful to compare the VTE risk in our patient population with that of general medical or surgical patients. However, audit registration in acute trusts without mental health provision was not feasible. We were unable to ascertain whether those identified as being at an increased risk of VTE went on to develop the condition.

Overall, our findings suggest that psychiatric in-patients are likely to be at increased risk of VTE. Older adults are most likely to be at risk. Further research is required to assess the risk posed by antipsychotics and physical restraint. Specific guidelines for VTE risk management in psychiatric patients and greater staff awareness of VTE risk are required.

## About the authors

**Natalie Ellis** is an undergraduate medical student at Cardiff University School of Medicine. **Carla-Marie Grubb** is an undergraduate medical student at Cardiff University School of Medicine. **Sophie Mustoe** is an undergraduate medical student at Guy's, King's and St Thomas' School of Medicine. **Eleanor Watkins** is an undergraduate medical student at Edinburgh University. **David Codling** is a Higher Trainee in Old Age and General Adult Psychiatry and a Darzi Leadership fellow. **Sarah Fitch** is an ST5 at Cardiff and Vale University Health Board. **Lucy Stirland** is a PsySTAR Clinical Research Fellow at the University of Edinburgh and a Higher Trainee in Old Age Psychiatry. **Munzir Quraishy** is an undergraduate medical student at Cardiff University School of Medicine. **Josie Jenkinson** is a Consultant Psychiatrist for Older People at Surrey and Borders Partnership, and co-opted mental health advisor for the 2018 revision of NICE guideline CG92. **Judith Harrison** is a Higher Trainee in Old Age Psychiatry and a Wellcome Trust GW4 Clinical Academic Training fellow at Cardiff University Brain Research Imaging Centre and the MRC Centre for Neuropsychiatric Genetics and Genomics.
